# Phase I/II trials of ^186^Re-HEDP in metastatic castration-resistant prostate cancer: post-hoc analysis of the impact of administered activity and dosimetry on survival

**DOI:** 10.1007/s00259-016-3543-x

**Published:** 2016-10-21

**Authors:** Ana M. Denis-Bacelar, Sarah J. Chittenden, David P. Dearnaley, Antigoni Divoli, Joe M. O’Sullivan, V. Ralph McCready, Bernadette Johnson, Yong Du, Glenn D. Flux

**Affiliations:** 1 Joint Department of Physics, The Institute of Cancer Research and The Royal Marsden Hospital NHS Foundation Trust, London, UK; 2 Division of Radiotherapy and Imaging, The Institute of Cancer Research and The Royal Marsden Hospital NHS Foundation Trust, London, UK; 3 Centre for Cancer Research and Cell Biology, Queen’s University Belfast, Belfast, UK; 4 Department of Nuclear Medicine, Brighton and Sussex University Hospitals NHS Trust, Brighton, UK; 5 Department of Nuclear Medicine and PET/CT, The Royal Marsden Hospital NHS Foundation Trust, London, UK

**Keywords:** Bone metastases, Prostate cancer, Radiopharmaceutical, Molecular radiotherapy, Dosimetry, Survival

## Abstract

**Purpose:**

To investigate the role of patient-specific dosimetry as a predictive marker of survival and as a potential tool for individualised molecular radiotherapy treatment planning of bone metastases from castration-resistant prostate cancer, and to assess whether higher administered levels of activity are associated with a survival benefit.

**Methods:**

Clinical data from 57 patients who received 2.5–5.1 GBq of ^186^Re-HEDP as part of NIH-funded phase I/II clinical trials were analysed. Whole-body and SPECT-based absorbed doses to the whole body and bone lesions were calculated for 22 patients receiving 5 GBq. The patient mean absorbed dose was defined as the mean of all bone lesion-absorbed doses in any given patient. Kaplan–Meier curves, log-rank tests, Cox’s proportional hazards model and Pearson’s correlation coefficients were used for overall survival (OS) and correlation analyses.

**Results:**

A statistically significantly longer OS was associated with administered activities above 3.5 GBq in the 57 patients (20.1 vs 7.1 months, hazard ratio: 0.39, 95 % CI: 0.10–0.58, *P* = 0.002). A total of 379 bone lesions were identified in 22 patients. The mean of the patient mean absorbed dose was 19 (±6) Gy and the mean of the whole-body absorbed dose was 0.33 (±0.11) Gy for the 22 patients. The patient mean absorbed dose (*r* = 0.65, *P* = 0.001) and the whole-body absorbed dose (*r* = 0.63, *P* = 0.002) showed a positive correlation with disease volume. Significant differences in OS were observed for the univariate group analyses according to disease volume as measured from SPECT imaging of ^186^Re-HEDP (*P* = 0.03) and patient mean absorbed dose (*P* = 0.01), whilst only the disease volume remained significant in a multivariable analysis (*P* = 0.004).

**Conclusion:**

This study demonstrated that higher administered activities led to prolonged survival and that for a fixed administered activity, the whole-body and patient mean absorbed doses correlated with the extent of disease, which, in turn, correlated with survival. This study shows the importance of patient stratification to establish absorbed dose–response correlations and indicates the potential to individualise treatment of bone metastases with radiopharmaceuticals according to patient-specific imaging and dosimetry.

## Introduction

Prostate cancer is the second most common cancer in men, with an estimated 1.1 million new cases worldwide in 2012 [1]. As the disease progresses to the castration-resistant stage, more than 80 % of patients develop bone metastases, which results in poor quality of life with an increase in skeletal pain and complications such as pathological fractures and spinal cord compression [2]. Systemic molecular radiotherapy with bone-seeking agents including ^32^P, ^89^Sr-chloride, ^153^Sm-EDTMP, ^186^Re-HEDP and ^188^Re-HEDP has been widely used in the management of pain [3].

The use of radiopharmaceuticals in cancer metastatic to bone is rapidly increasing. ^223^Ra-dichloride has been shown to improve survival compared to placebo [4] and radiolabelled anti-prostate-specific membrane antigen (PSMA)-targeted therapies show promise for diagnostic and therapeutic management of CRPC [5]; although long-term outcome data are not yet available for these agents. Repeated treatments and combination with chemotherapy and/or external beam radiotherapy have also demonstrated improved pain control [6–14]. Currently, most treatments are based on fixed levels of administered activity, which has resulted in a wide range of absorbed dose delivered to patients treated with bone-seeking radiopharmaceuticals [15–19]. A similar approach to that routinely used in external beam radiotherapy, whereby radiation doses delivered to tumours are safely maximised, would, in many cases, entail higher activity administrations, given the low levels of toxicity reported. This study investigated such potential for personalised treatments. To our knowledge, this is the largest study of bone lesion dosimetry to date. Although ^186^Re-HEDP is seldom used at present, the long-term follow-up data, methodology and results present valuable information for the design of future trials with bone-seeking radiopharmaceuticals.

^186^Re is a beta emitter with a half-life of 3.72 days, maximum beta energy of 1.07 MeV, average particle ranges of 1.1 mm in soft-tissue and 0.5 mm in bone, and a 9 % gamma ray emission at 137 keV that can be used for imaging. Chelated to hydroxyethylidene-diphosphonate (HEDP), ^186^Re-HEDP binds to hydroxyapatite crystals in bone and within 24 h approximately 70 % is excreted via the urine [20]. Several studies have examined the safety and efficacy of ^186^Re-HEDP in castration-resistant prostate cancer (CRPC) from administered activities ranging from 1110 to 3515 MBq. Average pain response rates from 50 to 89.5 %, and durations of pain relief for 6–10 weeks have been observed [21–34]. Toxicity was limited to mild transient myelosupression, with the platelet and white blood cell count nadir at 4 weeks [35] and a maximum tolerated activity of 2960 MBq was established [36].

To test the hypothesis that absorbed doses can be used as a predictive biomarker for outcome, a post-hoc analysis was performed using data from phase I and II clinical trials aimed at bone pain palliation in patients with metastatic CRPC treated with high levels of ^186^Re-HEDP and autologous peripheral stem cell transplantation. The impact of administered levels of activity and absorbed doses delivered on overall survival (OS) was assessed. A secondary aim was to investigate whether the absorbed dose delivered is associated with the extent of disease in order to study how this could determine the levels of administered activity.

## Materials and methods

### Patient population

Data are presented from phase I activity escalation and phase II fixed activity clinical trials conducted to examine the feasibility and safety profile of high administered activity levels of ^186^Re-HEDP and autologous peripheral blood stem cell transplantation for bone pain palliation in patients with metastatic CRPC [37, 38]. The patient cohort comprised 57 patients. Image data were obtained for 22 patients administered 5 GBq to allow dosimetry calculations. Eligibility criteria and population characteristics were the same in both trials and have been presented previously [37, 38]. A summary of patient characteristics relevant to the survival analysis are presented in Tables 1 and 2, including baseline prostate-specific antigen (PSA), alkaline phosphatase (ALP), haemoglobin, albumin and creatinine levels, administered activity and bone score/disease volume. The use of other therapies subsequent to ^186^Re-HEDP which have been proven to prolong survival such as docetaxel or ^223^Ra-dichloride is also indicated. All patients provided written informed consent to take part in the trials, which were approved by the Royal Marsden NHS Foundation Trust and The Institute of Cancer Research Ethics committee.

**Table 1:** Baseline characteristics for patients grouped according to outcome-oriented cut-point of the administered activity of 3.5 GBq

Characteristic	A <3.5 GBq (*n* = 12)	A >3.5 GBq (*n* = 45)
PSA (ng/ml) ^a^	76 (29–201)	81 (23–232)
ALP (U/l) ^a^	182 (137–538)	131 (88–275)
LDH (U/l) ^a*^	721 (615–944)	497 (412–595)
Haemoglobin (g/dl) ^a**^	11.4 (10.5–12.4)	12.9 (11.8–13.7)
Albumin (g/l) ^a^	39 (37–39)	38 (35–40)
Administered activity/no. (GBq) ^a^	3.1 (2.7–3.3)	5.0 (4.9–5.0)
Bone scan score ^b^	2 (1–3)	2 (1–3)
Docetaxel or ^223^Ra use following ^186^Re-HEDP/no. patients (%)	0 (0)	5 (11)
No.patients lost to follow-up (%)	0 (0)	2 (4)

^a^ Median and interquartile range. ^b^ Normal scan; 1: <6 metastases; 2: ≥6, <20 metastases; 3: >20 metastases but not superscan; 4: superscan or 75 % involvement of the skeleton. * *P* = 0.01. ** *P* = 0.009

**Table 2:** Baseline characteristics for patients grouped according to data-oriented cut-points of the patient mean (PMAD) and whole-body (WBD) absorbed doses

Characteristic ^a^	PMAD <19 Gy (*n* = 11)	PMAD >19 Gy (*n* = 11)	*P* value	WBD <0.28 Gy (*n* = 11)	WBD >0.28 Gy (*n* = 11)	*P* value
PSA (ng/ml)	28 (21–137)	122 (22–422)	0.3	28 (21–137)	122 (25–422)	0.2
ALP (U/l)	104 (72–127)	193 (98–439)	0.02	104 (72–127)	193 (98–336)	0.06
LDH (U/l)	506 (420–571)	512 (413–766)	0.3	506 (412–520)	564 (434–766)	0.09
Haemoglobin (g/dl)	12.8 (11.7–13.7)	12.9 (11.0–13.3)	0.6	13.1 (12.8–13.7)	11.7 (10.9–13.3)	0.1
Albumin (g/l)	36 (33–39)	35 (33–40)	0.7	37 (35–39)	34 (32–40)	0.3
Disease volume (ml)	81 (25 – 226)	280 (228–364)	0.002	81 (36–226)	299 (229–364)	0.0004
WBD (Gy)	0.25 (0.23–0.28)	0.37 (0.27–0.48)	0.01	0.25 (0.23–0.26)	0.38 (0.33–0.49)	0.0001
PMAD (Gy)	15 (10–17)	23 (22–25)	<0.0001	15 (11–19)	22 (18–25)	0.01

^a^ Median and interquartile range

### Data acquisition

Whole-body retention measurements were obtained using a 5-cm-diameter by 5-cm-thick collimated sodium iodide (NaI) scintillation detector 2 m above the patient [39]. Acquisition times were chosen to ensure Poisson noise levels were below 6 %. Anterior and posterior readings were measured within the limitations of catheterisation, which sometimes prevented measurements in the prone position. Up to 10 retention measurements were acquired over the 4 days following administration. To quantify the activity levels, the first measurement was obtained immediately after administration, before any activity was excreted from the patient.

Single-photon emission computed tomography (SPECT) scans of the thorax and pelvis were acquired at approximately 1, 4, 24, 48 and 72 h after administration using a Forte dual-head gamma camera (Philips Medical Systems, Reigate, UK) with a low-energy, high-resolution collimator. Patients were scanned with their arms down and the SPECT images comprised 64 projections (20 s per projection) per head in 128 × 128 matrices with a 4.67-mm voxel size. Energy windows with widths of 20 and 7 % were centred on the main ^186^Re peak at 137 keV and for scatter correction just below the peak at 119 keV, respectively. CT scans were not available. SPECT data were reconstructed using filtered-back projection (FBP) and pre-filtered with a Butterworth filter of order 2 and cut-off 1. Images were scatter-corrected using the dual-energy window method (DEW) and attenuated-corrected using a uniform linear attenuation coefficient of 0.142 cm^−1^ within elliptical patient outlines. A whole-body scan acquired with a scan speed of 12 cm/min, 1–2 days following administration, confirmed that the majority of lesions were seen within the field of view of the pelvic and thoracic SPECT scans.

### Dosimetry

To compensate for the underestimation of the activity in the bone lesions due to partial volume effects, a recovery curve was obtained using 11 cylindrical phantoms (1 × 1, 1.5 × 1.5, 2 × 2, 3 × 3, 4 × 4, 5 × 5, 1 × 10, 2 × 10, 3 × 10, 4 × 10 and 5 × 10 cm). Volumes ranged from 0.8 to 196 ml and contained ^186^Re with an average activity concentration of 2.20 (±0.05) MBq/ml. Each of the cylinders was placed in a large water-filled cylinder of 19 cm diameter and 11 cm height, scanned, and reconstructed using the same parameters as for the patient data. Optimum threshold values to recover the physical phantom volumes were obtained.

Whole-body absorbed doses were calculated from the retention data with corrections applied for individual patient mass. Activity quantification for imaged lesions was achieved by volume of interest analysis carried out on a HERMES workstation (Hermes Medical Solutions, Stockholm, Sweden) using thresholds and sensitivity factors obtained from phantom studies. Absorbed dose distributions were obtained from the convolution of a voxelised cumulated activity distribution and a voxel S-value kernel for ^186^Re with 4.67-mm voxels in a soft-tissue density medium. Cumulated activity distributions were derived from the integration of time-activity curves obtained from the co-registered sequential SPECT scans on a voxel by voxel basis. For the uptake phase, it was assumed that the activity at the time of administration was zero and linearly increased to the first scan time. For the last phase, exponential decay with a physical half-life was assumed from the last scan point to infinity to avoid any bias introduced by registration errors and redistribution of uptake at the voxel level. Trapezoidal integration or mono-exponential fitting were used for the intermediate phases depending whether the activity increased or decreased over time, respectively. The absorbed dose kernel was generated using an in-house application developed using the EGS++ class library within the general purpose EGSnrc Monte Carlo (MC) code [40, 41], which was previously validated [42]. The ^186^Re decay spectra used in the simulations was obtained from the RADTABS software [43].

Metastatic bone lesions were outlined on the absorbed dose distributions on the HERMES workstation using volume-dependent thresholds obtained from the phantom studies. The mean absorbed dose delivered was calculated for each metastatic lesion. For any given patient, the patient mean absorbed dose was defined as the mean of the individual lesion absorbed doses and the disease volume was defined as the sum of all the lesion volumes identified. The relationships between the disease volume and the whole-body and patient mean absorbed doses were investigated. The relationship between the disease volume and baseline levels of ALP was also studied.

### Response

Baseline and follow-up bone scans were not available to assess treatment response. Following intravenous administration of ^186^Re-HEDP, biochemical measurements that included PSA and ALP were performed weekly before and after treatment until progression. Correlations between the patient mean absorbed dose and maximum change in PSA and ALP levels were investigated, where the maximum change was calculated as the difference between baseline and nadir relative to baseline level.

### Survival

Survival was determined from the date of ^186^Re HEDP administration until death from any cause, until the last follow-up or until the start of treatment with docetaxel or ^223^Ra-dichloride. The impact of the administered activity on OS was studied in the cohort of 57 patients. The sub-cohort of 22 patients, for whom dosimetry was performed, was used to analyse the impact of the patient mean and whole-body absorbed doses, volume of disease, and baseline levels of ALP and PSA on the OS.

### Statistical analysis

Mean (± standard deviation) and confidence intervals (CI) were used to describe normally distributed continuous variables and the median with range otherwise. Regression analysis and Pearson’s correlation coefficient were used to assess linear relationships between two variables. The disease volume and ALP levels were not normally distributed and, therefore, a natural logarithm transformation was applied for the regression analysis. Differences in PSA and ALP response were analysed by patient subgroups that included the patient mean absorbed dose. Statistical significance was assessed using Fisher’s exact test to compare the proportion of patients with declines ≥50 % in PSA and ALP levels for mean absorbed doses below and above the median. Differences between baseline characteristics were evaluated by the *t* test or Mann–Whitney test.

Survival curves were estimated by the Kaplan–Meier method and comparisons between groups were made with the log-rank test and hazard ratios (HR) with 95 % CI. The phase I study showed a statistically significant correlation between PSA response (≥50 % decrease for ≥4 weeks) and activity administered (*P* = 0.015, two-sided Fisher’s test), with a response rate of 20 % in patients that received more than 3.5 GBq of ^186^Re-HEDP [38]. This outcome-oriented cut-point for the administered activity level was used to divide the entire patient cohort into two groups to assess the impact of administered activity on OS. Survival was also studied in the 22-patient sub-cohort stratified according to dichotomised values of the disease volume, the patient mean absorbed dose, the whole-body absorbed dose, and baseline levels of ALP and PSA. A data-oriented cut-point based on median values was chosen for these variables as a biologically driven approach was not available and this provided equal sized groups. The effect of disease volume, and whole-body and patient mean absorbed doses on OS was also assessed by using multivariable Cox regression analysis. Two-sided exact *P* values below 0.05 were considered statistically significant.

## Results

### Dosimetry

**Fig. 1:**
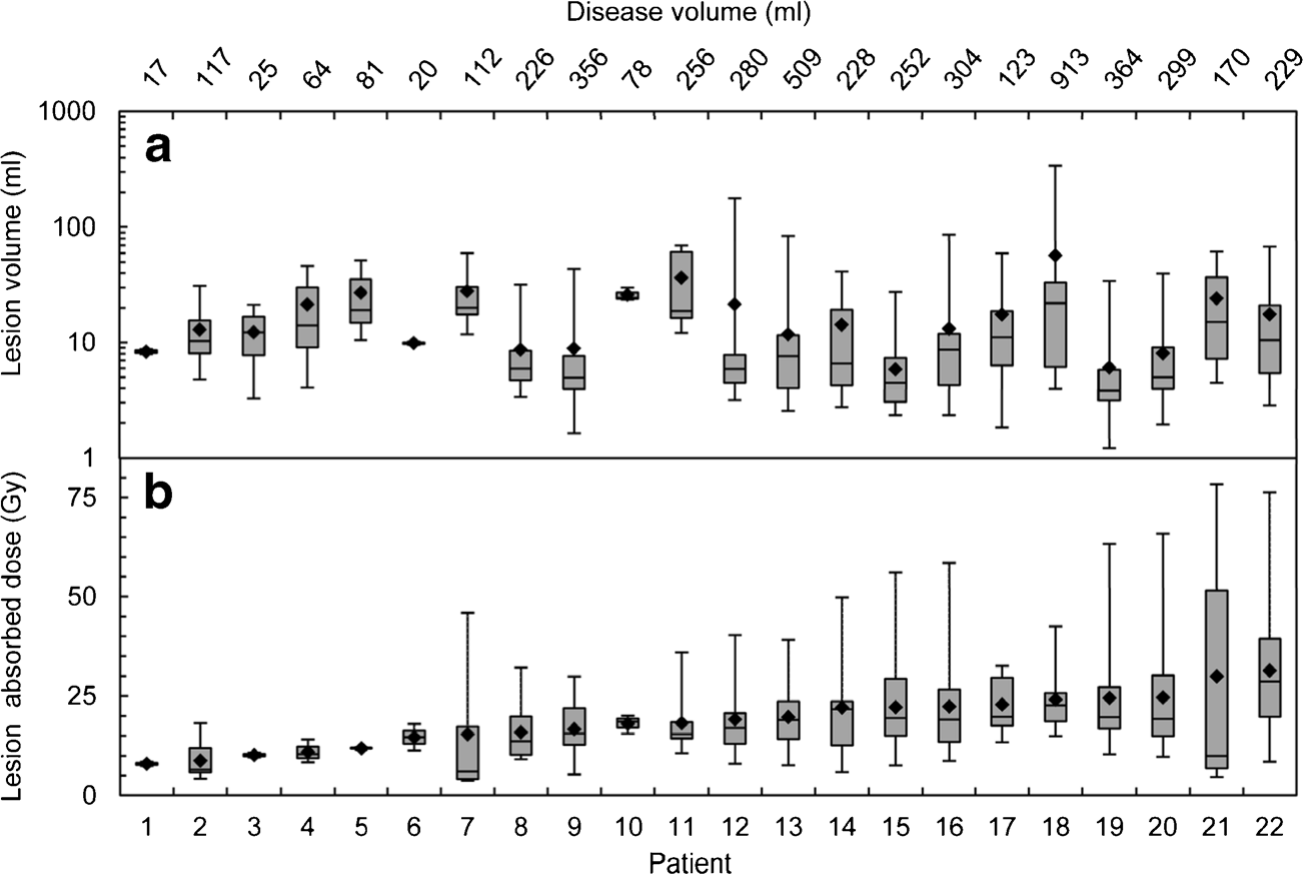
Box plot representing the intra- and inter-patient variability of the lesion volumes (**a**) and absorbed doses (**b**) for the sub-cohort of 22 patients. For any given patient, the whiskers display the minimum and maximum values. The patient mean absorbed dose and mean lesion volume are shown with full *diamond* symbols

A 40 % threshold recovered volumes down to 10 ml and thresholds of 58 and 71 % were necessary to recover the 1.5 × 1.5 and 1 × 1 cm cylinders, respectively. A total of 379 metastatic lesions were identified in 22 patients that received an administered activity of 5 GBq of ^186^Re-HEDP, with a median of 11 lesions (range: 2–60) and 227 ml of disease volume (range: 17–913 ml) per patient (Fig. 1a). The mean whole-body absorbed dose was 0.33 (±0.11) Gy (95 % CI: 0.28–0.38 Gy). Individual lesion absorbed doses ranged from 4 to 78 Gy (Fig. 1b). The patient mean absorbed dose ranged from 8 to 31 Gy with a mean of 19 (±6) Gy (95 % CI: 16–22 Gy) across the 22 patients. Median values of the whole-body and patient mean absorbed doses across all patients of 0.28 and 19 Gy, respectively, were used for the grouped survival analysis.

**Fig. 2:**
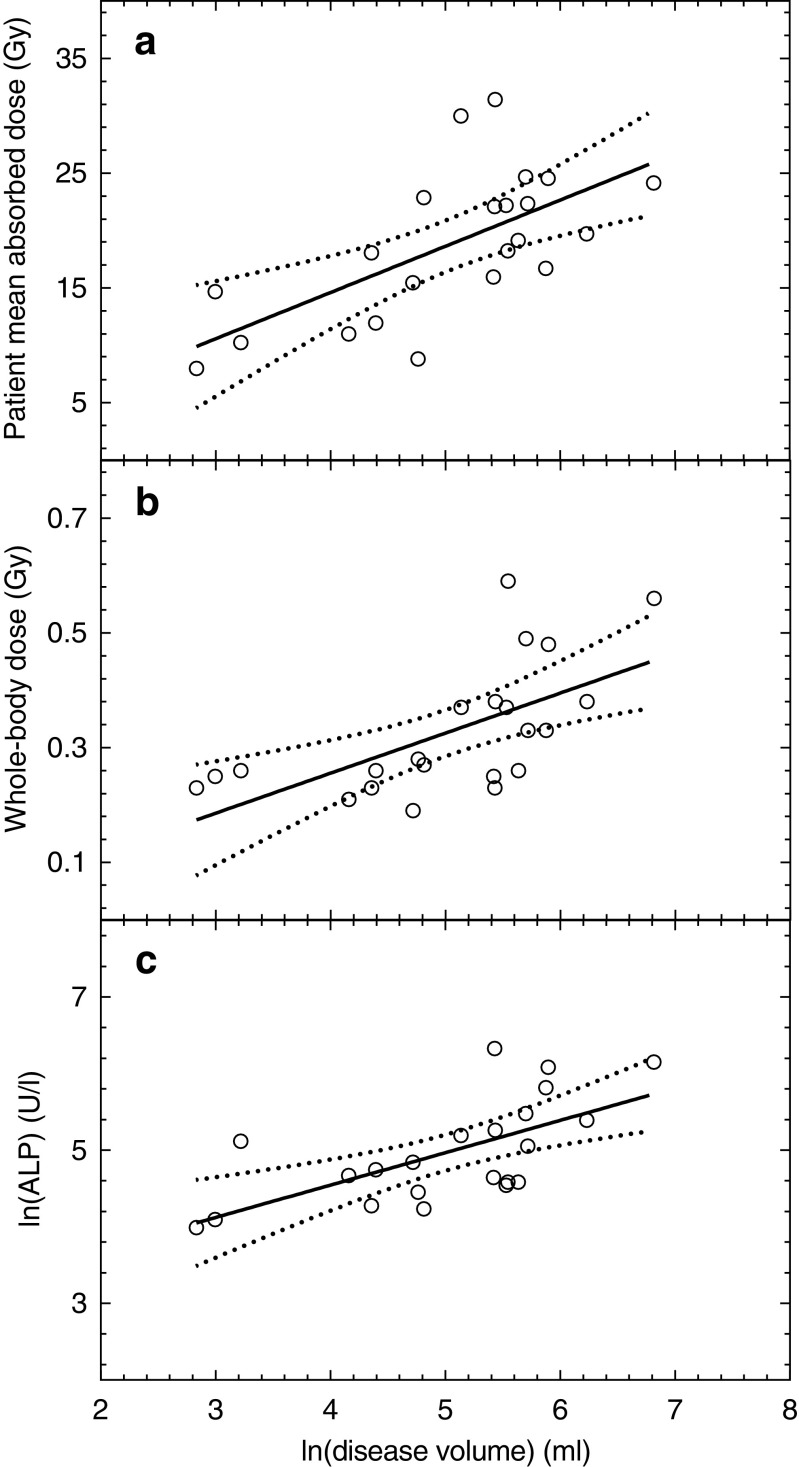
Relationships of the patient mean absorbed dose (**a**), the whole-body absorbed dose (**b**) and the baseline level of ALP (**c**) with the disease volume variable for the sub-cohort of 22 patients. The *dotted *lines represent the 95 % confidence intervals

The patient mean absorbed dose (*r* = 0.65, *P* = 0.001) and the whole-body absorbed dose (*r* = 0.63, *P* = 0.002) showed positive correlations with the disease volume, shown in Fig. 2a and b.

### Response

**Fig. 3:**
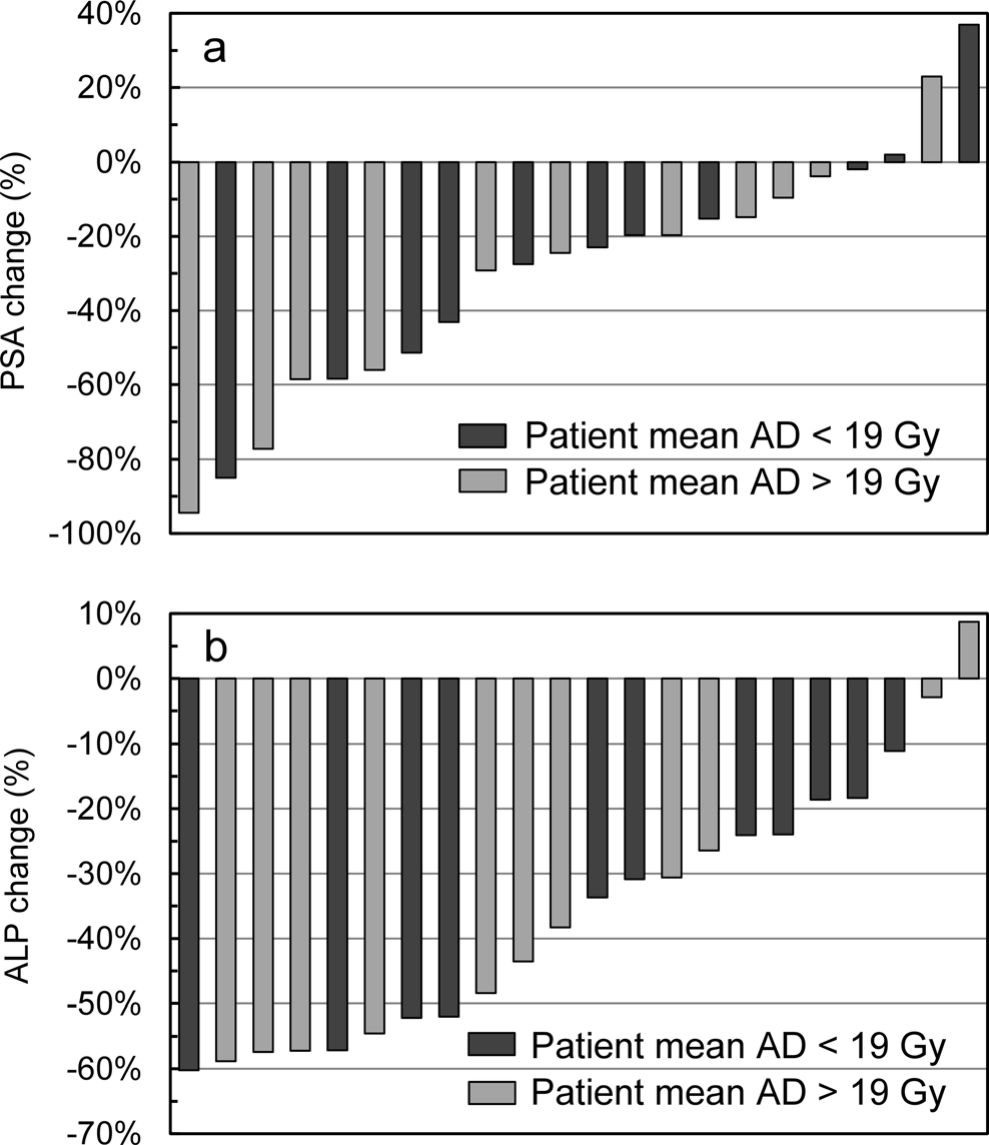
Maximum change in PSA (**a**) and ALP (**b**) levels from baseline in the subgroup of 22 patients. Patients that received a patient mean absorbed dose (AD) below and above 19 Gy are shown in *dark* and *light grey*, respectively

The maximum change in PSA and ALP levels for each patient was grouped according to the patient mean absorbed dose, shown in Fig. 3. A total of 8 out of 22 patients had a decrease ≥50 % in ALP and 7 out of 22 patients had a decrease ≥50 % in PSA levels. However, the reduction of PSA (*P* = 1.0) and ALP (*P* = 1.0) levels were not related to the patient mean absorbed dose.

### Survival

Two patients were lost to follow-up and, therefore, censored at the last point of contact. Five patients were censored at the time of treatment with docetaxel or ^223^Ra-dichloride to avoid bias due the survival benefit associated with these therapies. The median OS from the time of treatment was 17.5 months for the entire patient cohort, with estimated 1- and 2-year survival rates of 72 % (95 % CI: 58–82 %) and 31 % (95 % CI: 19–43 %), respectively. The median OS for the sub-cohort of 22 patients was 18.5 months.

**Fig. 4:**
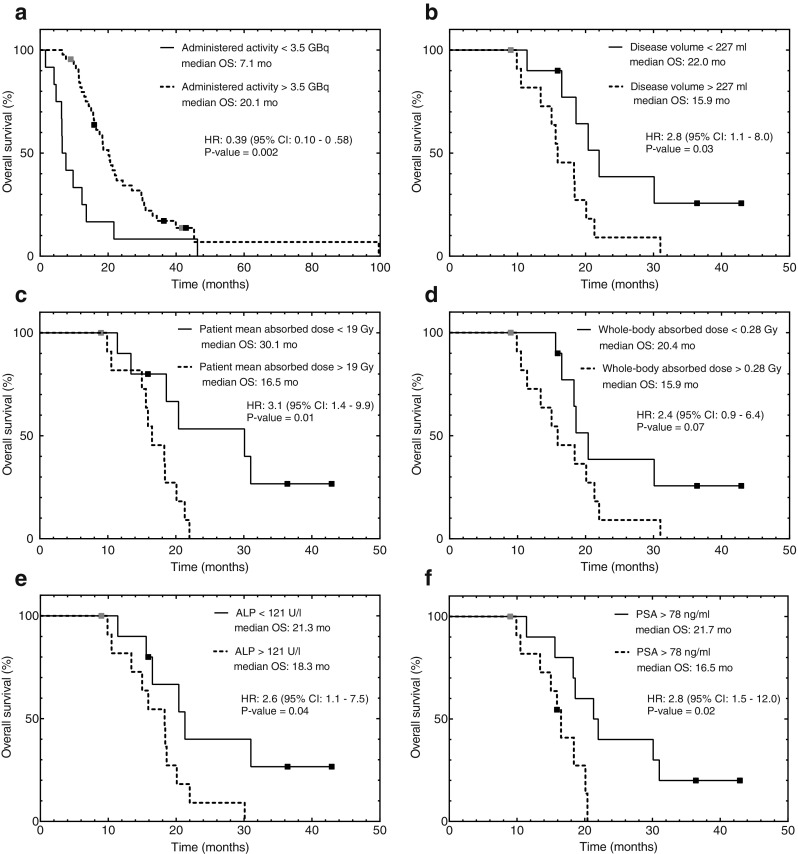
Kaplan–Meier estimates of overall survival for the cohort of 57 patients divided according to administered activities below and above 3.5 GBq of ^186^Re-HEDP (**a**); and for the sub-cohort of 22 patients grouped according to median values of disease volume (**b**), patient mean absorbed dose (**c**), whole-body absorbed dose (**d**), and baseline levels of ALP (**e**) and PSA (**f**). Five patients were censored at the time of treatment with ^223^Ra or docetaxel (*black squares*) and two patients were censored at the last known follow-up (*grey squares*)

Kaplan–Meier curves and HR for the univariable grouped analyses are shown in Fig. 4. The grouped analysis of the 57 patients showed that administered activities above 3.5 GBq were associated with a 61 % death risk reduction (HR, 0.39; 95 % CI, 0.10–0.58; *P* = 0.002), with a median OS of 20.1 months as compared to 7.1 months in the lower activity group. Table 1 shows statistically significant differences in baseline levels of lactate dehydrogenase (LDH) and haemoglobin. However, according to Halabi’s nomogram [44], these differences in prognostic factors cannot explain the 13 months of survival benefit observed.

In the sub-cohort (Fig 4b to f), median OS of 16.5 and 30.1 months was observed in patients receiving patient mean absorbed doses above and below 19 Gy (HR, 3.1; 95 % CI, 1.4–9.9; *P* = 0.01), and of 15.9 and 20.4 months for patients receiving whole-body absorbed doses above and below 0.28 Gy (HR, 2.4; 95 % CI, 0.9–6.4; *P* = 0.07), respectively. A longer OS (22.0 vs 15.9 months) was observed in patients with a smaller disease volume (HR, 2.8; 95 % CI, 1.1–8.0; *P* = 0.03). Median OS values of 21.3 and 18.3 months were obtained for baseline ALP levels below and above 121 U/l (HR, 2.6; 95 % CI, 1.1–7.5; *P* = 0.04), and 21.7 and 16.5 months for baseline PSA levels below and above 78 ng/ml (HR, 2.8; 95 % CI, 1.5–12.0; *P* = 0.02), respectively. The Cox regression analysis including the disease volume (HR, 5.7; 95 % CI, 1.7–18.4; *P* = 0.004), whole-body absorbed dose (HR, 0.003; 95 % CI, 0.0–2.0; *P* = 0.08) and patient mean absorbed doses (HR, 1.0; 95 % CI, 0.9–1.1; *P* = 0.9); showed that only the disease volume retained its significance in predicting OS. Differences in baseline characteristics between groups divided according to patient mean and whole-body absorbed doses (Table 2) were significant for ALP levels and disease volume, in addition to the expected cut-point variables. This was expected as the absorbed doses are correlated with the disease volume (Fig. 2a and b), which, in turn, was positively correlated with ALP levels (*r* = 0.65, *P* = 0.001; Fig. 2c).

## Discussion

This post-hoc analysis found a significantly longer OS associated with administered activities above 3.5 GBq and showed that patients with a higher disease volume received higher whole-body and patient mean absorbed doses.

A total of 379 lesions were identified in 22 patients. Inter-patient comparisons showed a range of absorbed doses delivered from fixed administered activities with a mean patient mean absorbed dose of 19 (±6) Gy across the 22 patients, in agreement with a former feasibility study [45]. A large heterogeneity in the absorbed dose delivered to individual lesions was observed, ranging from 4 to 78 Gy for administrations of 5 GBq. These are in close agreement with those calculated by Israel et al. [46] using quantitative sequential SPECT imaging, with lesion absorbed doses ranging from 0.36 to 8.03 Gy from administered activities of 1380–1850 MBq. Maxon et al. reported a significantly higher lesion mean absorbed dose of 40 Gy from 1.225 GBq, although these calculations were not based on SPECT imaging [22]. Whole-body absorbed doses of 0.04–0.12 mGy/MBq from 5 GBq and peripheral stem cell transplantation were obtained in this study, which are comparable to 0.03–0.18 mGy/MBq from 1.251 to 4.144 GBq reported by Graham et al. [47].

Accurate calculation of the absorbed doses delivered to bone lesions is challenging. Uptake of bone-seeking radiopharmaceuticals does not directly reflect the tumour volume, as it depends on the osteoblastic activity. The spatial resolution of available clinical systems does not allow the heterogeneous distribution of uptake at the microscopic level to be determined. Therefore, it was assumed that the uptake was indicative of the extent of the bone lesion, similar to other studies performing bone lesion dosimetry [15–19]. More refined dosimetry calculations would account for differences in the uptake distribution at the microscopic level. Samaratunga et al. developed a heterogeneous dosimetry model based on Monte Carlo radiation transport simulations, histomorphometry and autoradiography analysis of the ^186^Re-HEDP deposition in bone lesions [48]. They found that the uniform uptake model underestimates the absorbed dose to osteoblastic and mixed lesions by factors of up to 1.84 and 1.39, respectively, with similar results for osteolytic lesions. However, this study was based on bone biopsies from a single metastatic site and its use is, therefore, limited. The methodology presented here is more practical in a routine clinical setting. The relative absorbed doses obtained for the large number of lesions identified are not expected to be greatly affected by the assumptions made. Nonetheless, this highlights the necessity for standardisation of imaging protocols and dosimetry methodology to enable the comparison of treatments. The present availability of multimodality and hybrid imaging has the potential to provide improved image quantification and dosimetry. Furthermore, both therapeutic and diagnostic radiopharmaceuticals that show comparable mechanisms of uptake may enable adaptive treatment planning for repeated treatments.

For a fixed level of administered activity, higher whole-body and patient mean absorbed doses were delivered in patients with a larger disease volume. Patients with more metastases were expected to receive a higher whole-body absorbed dose, as a larger proportion of the administered activity is retained in patients with more lesions. However, it had not been anticipated that the lesion mean absorbed dose would also be higher. Previous studies have shown a positive linear correlation between the bone uptake as a percent of the administered activity and the number of metastatic bone lesions for ^153^Sm-EDTMP and ^186^Re-HEDP due to the lower urinary excretion in patients with a large disease volume [49, 50] . Our study found no correlation between the absorbed doses and the renal function, determined from the glomerular filtration rate (GFR) measured before treatment using the ^51^Cr-EDTA clearance rate. Farhanghi et al. concluded that the wide ranges in renal excretion could not be attributed to variations in renal function, since all the patients had normal creatinine levels [49]. Using ^89^Sr, Blake et al. found that low values of renal clearance correlated with the elevation of serum parathyroid hormone and nephrogenous cyclic adenosine monophosphate levels, which, in turn, correlated with the disease volume [51]. An increase in bone uptake could also occur in response to an increase in bone mineral turnover as the disease progresses and new metastases develop. All these biomarkers are likely to play a significant role in the absorbed doses delivered. Further studies with larger patient cohorts are needed to fully elucidate the mechanisms of uptake of bone-seeking radiopharmaceuticals and to establish absorbed dose response correlations.

This study has demonstrated that other factors may be useful to predict response to treatment with molecular radiotherapy. A significantly longer OS was associated with administered activities above 3.5 GBq of ^186^Re-HEDP (7.1 vs 21.2 months, *P* = 0.002). A similar survival advantage at higher administered activities has also been reported in patients treated with anti-PSMA ^177^Lu-J591, with median OS of 11.9 and 21.8 months (*P* = 0.03) for administered activities of 2.4 and 2.6 GBq, respectively [52]. Our study also found that a shorter survival was associated with higher absorbed doses due to the positive correlation between absorbed dose and the disease volume. The multivariable analysis showed that patient mean absorbed dose was not correlated with survival when corrected for the disease volume, which is a well-established prognostic marker of survival in metastatic prostate cancer [53–55].

From the theragnostic point of view, dosimetry can be indicative of functional aggressiveness of the disease in addition to being a marker of response, since higher absorbed doses are associated with a higher probability of cell kill. The results found in this post-hoc analysis suggest that the administered activity and the absorbed doses delivered to the whole body and the bone lesions have potential use as predictive biomarkers, which warrant further studies. The OS benefit observed for higher administered activities would be limited by red marrow toxicity. Buffa et al. developed a model to predict the whole-body dose prior to treatment with ^186^Re-HEDP based on individual patient biochemical and physiological parameters, finding a strong correlation between whole-body absorbed dose and platelet and white blood cell count toxicity [56]. Such models could be extended to incorporate information from bone metastases markers to provide a method of personalised treatment.

## Conclusion

A statistically significant survival benefit was observed for higher administered activities. The wide range of absorbed doses delivered from a fixed administered activity was indicative of the heterogeneity of the disease. Similar studies with other bone-seeking radiopharmaceuticals are required to fully understand the role of the different biomarkers in the absorbed doses delivered and to determine whether quantitative imaging and dosimetry can lead to individualised treatment planning. Given the correlation of disease volume with absorbed dose, activity escalation trials with patients stratified according their skeletal tumour burden could help to identify in more detail absorbed dose response correlations. The implementation of imaging and dosimetry will enable the development of novel methods of sequential administration of radiopharmaceuticals and adaptive treatment planning in patients with CRPC treated with radiopharmaceuticals.
